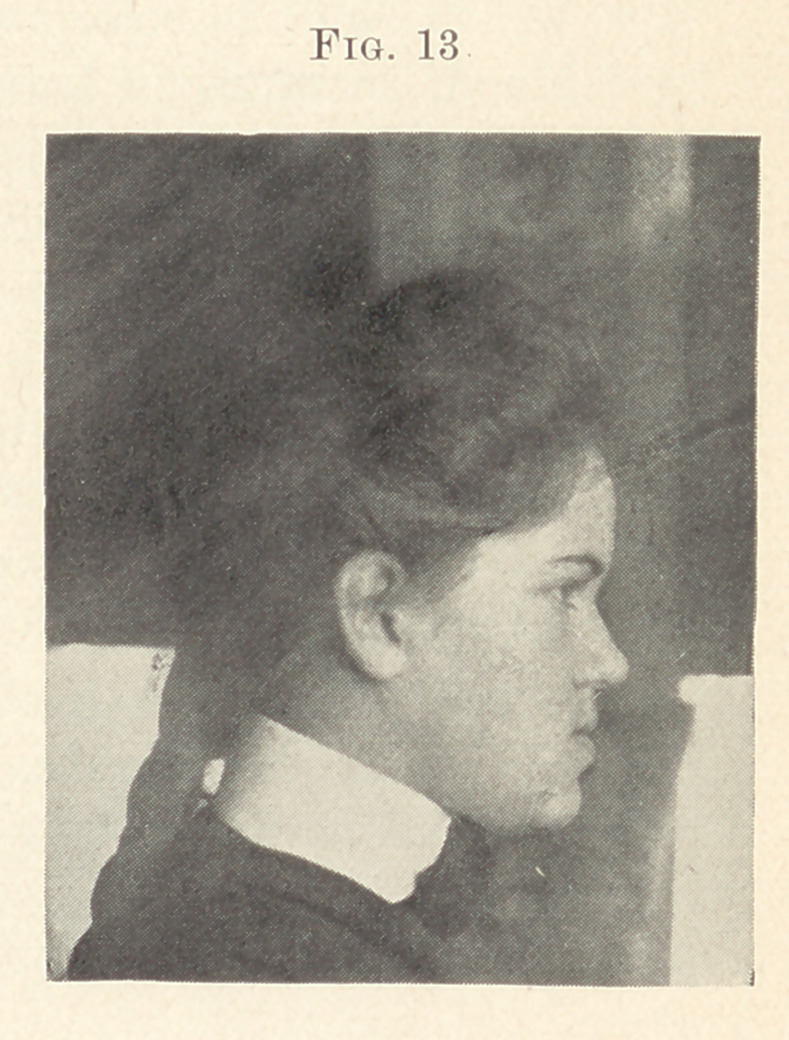# Original Investigation Not a Necessary Quality of the Teacher

**Published:** 1901-03

**Authors:** C. M. Wright

**Affiliations:** Cincinnati, Ohio


### ORIGINAL INVESTIGATION NOT A NECESSARY QUALITY OF THE TEACHER.

BY DR. C. M. WRIGHT, D.D.S., CINCINNATI, OHIO.

THE president of a great university has given expression to the opinion that university professors should be original investigators. This might be taken as a general proposition, embracing professors of the various branches of science, like chemistry, biology, physics, and psychology, and refer to special laboratory experiments; and it might include the more literary branches like philology, history, and political economy, and refer to studies of an original character. In each case the idea expresses the desirability of a special distinction or reputation for the study of some small part of science and the adding of some small fragment of fact to the general fund of human knowledge. The translation of some work from an ancient or modern language, the digging out of some obscure hieroglyphic,

Fig. 10.

Fig. 11.

Fig. 12.

Fig. 13

the announcement of some startling doctrine in history or art or politics, or perhaps the editing of a text-book, might be considered an equivalent; but some of these special and distinctive things the professor must do to satisfy the demand of this university president.

A few years ago, in an address on the occasion of the opening of a new building for the Ohio College of Dental Surgery, I expressed thoughts differing entirely from the above notion. Subsequent thinking on the subject has not changed my opinion. I then claimed that the teacher need not be an "investigator," a writer, nor a great artist. I claimed that he should be, in the highest sense, simply a *teacher*. He occupies the place of middleman, between the investigator, the fact-hunter, the learned and patient gatherer of accepted facts and fancies in the various fields of knowledge, on the one hand, and the *student* or pupil on the other. He searches through the goods of the manufacturer and collector, selects what he deems good and proper for his customer, the student, and then exhibits them in his best style or art, the most necessary quality of which is his method of presenting and displaying the goods.

Teaching is a special art, a most important one. It is an art which has occupied the deepest thought of profound philosophers from remote times,—witness Descarte and the "Venerable" Bede. I am almost tempted to call it the art of arts, for it stands between the world, nature—all things seen and unseen—and the mind of man. It is only by the skill of the teacher that facts or doctrines can be digested and prepared for reception and assimilation by the student. In great universities, like some of those in the capitals of Europe and America, the original investigator should have his place and his title of professor, but he should be on furlough most of the time. The university furnishes the air suitable for his health, and his connection and association add an element to this same air which makes it favorable to students and the teaching corps. There are reflex benefits easily traced. From the advertising or business stand-point the work of these scientist-professors is also profitable, for they win distinction and fame for the university and draw students to its halls. Now, the teacher or the teaching professor plays a different *rôle*. He culls the flowers from the investigator's conservatory, arranges them into bouquets, groups them into arches and wreaths, and presents them to the developing minds of the students; and they, according to their own and the teacher's ability, and according to the art of the teacher, gradually grasp the meaning and the beauty of the flowers and their places in the harmony of the knowledge which is power.

A teacher may be a special investigator, and an investigator may be a teacher, but my point is that they are distinct organs in the body, with different functions, and yet with an interdependence similar to that existing among different tissues of an organism. The teacher need not devote days and weeks in his laboratory, with reagents, microtomes, and microscopes, studying, let us say, for example, a villus in the alimentary canal. He needs only to be awake to grasp the fact that the epithelia of the villus may be possessed of a peculiar nucleus, or that the lining tissue of the radicle is suggestive of some peculiar function, which facts may have taken months of investigation, after years of special training on the part of the scientist. And then the teacher must be able to emphasize points, attract attention, elaborate, simplify, illustrate, and become enthusiastic over the fact, so that a hundred students may have this little seed sunk into their cortices, that it may spring up later into knowledge.

I once knew a Yankee tutor who came out West and established a classical and scientific school for boys. The scholars of the city sneered at the pretensions and attainments of the young man. "He has no diploma or degree. He was only an under-tutor in a Massachusetts academy. He is a charlatan in education, and cannot succeed." The young man used to reply good-humoredly, "I am a teacher. I have taught school since I was fifteen years of age."

I attended a banquet, the other day, of the alumni of the "Academy" established by this teacher,—the tenth annual banquet since his death and the closing of the academy,—and there met some of the most learned professional men, prominent politicians, business men, and distinguished teachers of the city; and in the post-prandial addresses it was noticeable that supreme credit was accorded the dead man as "the successful teacher." My own experience with and reflections about the methods of this teacher placed me in accord with the universal opinion, for algebra, geometry, physical geography, and other branches taught by him were so illuminated in their presentation by the genius of the man that they have ever remained as clearly defined stepping-stones in my own pathway of development; and yet this man was in no sense a mathematician or a geographer.

Another example: A German applied for the position of teacher of English in a Swiss city school. "You are not an Englishman; how can you teach English?" asked the board. "You did not ask for an Englishman, but a teacher of English, and I am a *teacher.*" He emphasized the "*teacher.*" He received the appointment, and proved by years of service and satisfaction that he had the correct idea.

One more picture. One of the most distinguished scientists of Cincinnati, a chemist, an astronomer, a mathematician,—referred to, by the way, in Professor Lloyd's "Etidorpha,"—was professor of chemistry in medical and dental schools of this city many years ago. He was appointed on account of his distinction in knowledge and world-wide scientific reputation, yet, with the deepest reverence for his work, reputation, and personality, I think I can honestly say that his students in the aforesaid colleges did not grasp, in the least, the subjects he tried to teach. He could not adapt himself to the plane of understanding of the ordinary student. He was not a teacher. This man was an honor to any institution with which he might be connected—but he should have been kept in his laboratory at the expense of the school.

Some of the great ones in the art of dentistry, whose works are masterpieces in operative or prosthetic dentistry, cannot teach.

Great lecturers, writers, and critics on art, whose words become inspirations and axioms on perspective, or color, or metre, may not be able to draw or paint, or strike a note. They are teachers. The art of teaching is different from the art of investigating or doing.

## Figures and Tables

**Fig. 10. f1:**
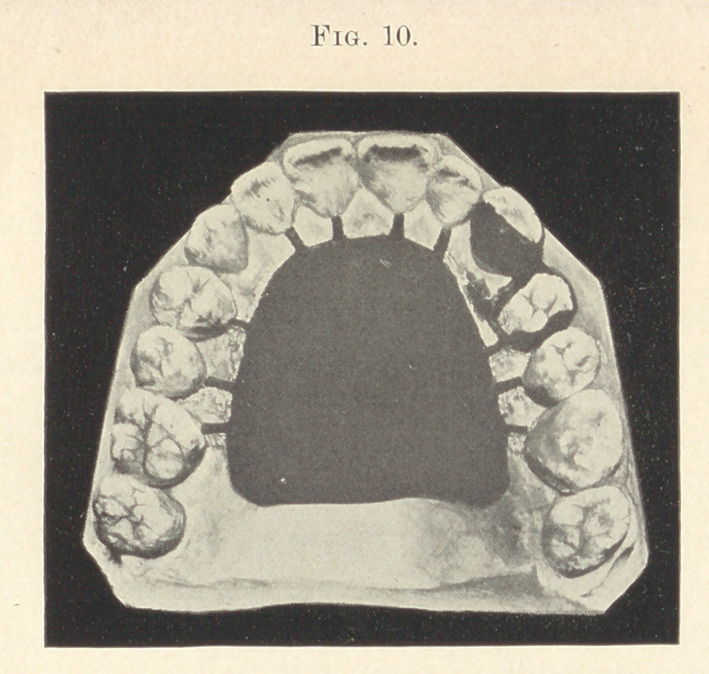


**Fig. 11. f2:**
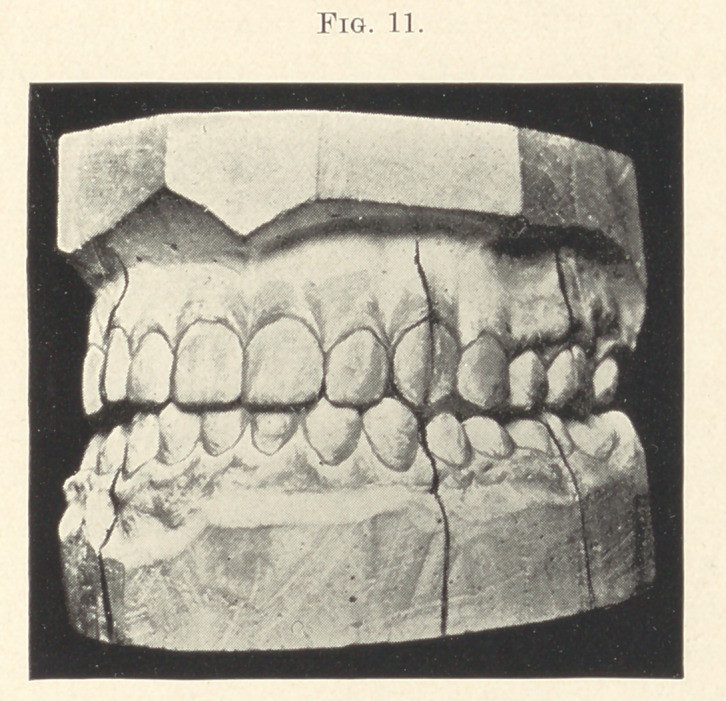


**Fig. 12. f3:**
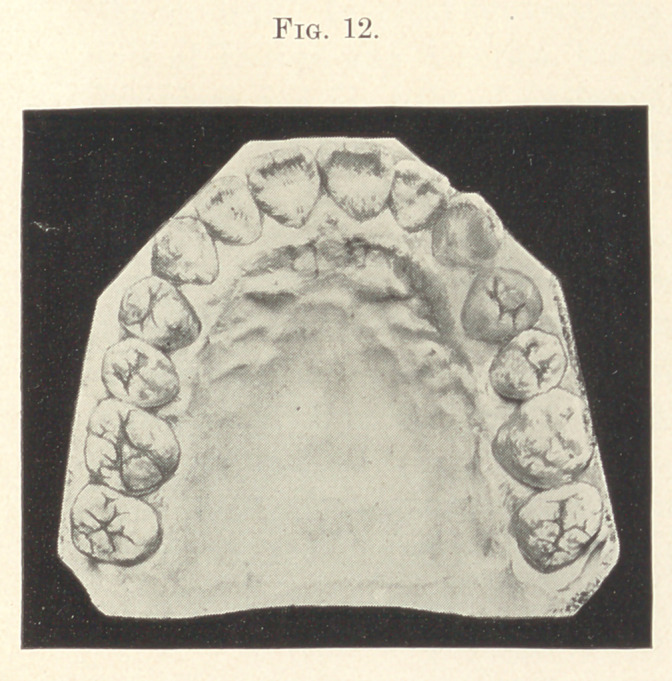


**Fig. 13. f4:**